# Upregulation of Hsp90-beta and annexin A1 correlates with poor survival and lymphatic metastasis in lung cancer patients

**DOI:** 10.1186/1756-9966-31-70

**Published:** 2012-08-28

**Authors:** Rong Biaoxue, Jiang Xiling, Yang Shuanying, Zhang Wei, Cai Xiguang, Wang Jinsui, Zhang Min

**Affiliations:** 1 Department of Respiratory Medicine, Second Affiliated Hospital, Xi’an Jiaotong University, 157, Xi 5 Road, Xi’an, 710004, People's Republic of China; 2 Department of Oncology, Weinan Central Hospital, Weinan, China; 3 Department of Respiratory Medicine, People’s Hospital of Gansu Province, Lanzhou, China; 4 Department of Pathology, People’s Hospital of Gansu Province, Lanzhou, China

**Keywords:** Lung cancer, Hsp90-beta, Annexin A1, Survival, Lymphatic metastasis, Biomarker

## Abstract

**Background:**

Hsp90-beta and annexin A1 were investigated as prognostic factors because of their apparent association with tumorigenesis. However, the effect of Hsp90-beta and annexin A1 in lung cancer remains poorly understood. The expressions of Hsp90-beta and annexin A1 in lung cancer and normal lung specimens were examined, and the relationships with respect to the clinico-pathological features and patient survival in lung cancer were analyzed.

**Methods:**

The expression levels of Hsp90-beta and annexin A1 were examined using immunohistochemistry, *in-situ* hybridization, and Western blot.

**Results:**

Lung cancer tissues exhibited higher expression levels of Hsp90-beta and annexin A1 than the normal tissues (p < 0.05), and the expression levels of the markers were significantly associated with the pathological grade and lymphatic invasion of lung cancer (p < 0.05). Moreover, the upregulation of Hsp90-beta and annexin A1 correlated with decreased survival (p < 0.05).

**Conclusion:**

The upregulation of Hsp90-beta and annexin A1 were associated with poor post-surgical survival time and lymphatic metastasis of lung cancer patients. Moreover, the high expression of the markers was an independent predictor of poor outcomes.

## Background

Lung cancer is one of the leading causes of deaths globally. An estimated 222,520 new cases of lung cancer were reported in 2010, which accounted for approximately 15% of cancer diagnoses. This disease accounts for a higher percentage of deaths than any other cancer in both men and women. An estimated 157,300 deaths, which accounted for approximately 28% of all cancer deaths, were reported in 2010 [1]. The high rate of mortality is most likely attributed to early metastasis that causes malignant cells to spread to various tissues including bone, brain, and liver tissues. The early detection of cancer leads to a better prognosis for reduced mortality and morbidity. The advent of new and emerging molecular, genetic, and imaging technologies has broadened the possible strategies for early detection and prevention. However, the decrease in mortality should be supported by clinical evidence.

Proteomics has recently emerged as a powerful technology to identify differential protein expressions associated with cancer development and progression. New strategies that facilitate proteomic analysis by mass spectrometry (MS) have been introduced for biomarker discovery research. We have previously identified the upregulation of annexin A1 and Hsp90-beta in 16 human bronchial epithelial (HBE) cell lines compared with the NCI-A549 and NCI-H446 with two-dimensional liquid chromatography-tandem mass spectrometry (2D-LC-MS/MS). However, the origin and natural expression of the clinical specimens of lung cancer remain unknown. Annexin A1, an intracellular protein that can bind calcium and phospholipid, have several important functions in cell proliferation, apoptotic regulation, apoptotic cell phagocytosis, and carcinogenesis [2]. Several findings concerning its role in tumorigenesis are controversial. Annexin A1 expression has been shown to be down regulated in several cancers such as esophageal, prostate, breast, and larynx cancers [3,4]. However, this marker was upregulated in several other cancers such as pancreatic and hepatocellular carcinoma as well as in several types of breast cancers [5,6]. Heat shock protein 90 (Hsp90) is a highly abundant and evolutionarily conserved protein in eukaryotic cells. Five Hsp90 isoforms have been identified to date, which include the two major cytoplasmic isoforms, namely, Hsp90-alpha and Hsp90-beta [7]. Hsp90-beta is probably involved in long-term cellular adaptation, and higher levels of Hsp90-beta are involved in normal cellular functions, such as the maintenance of the cytoarchitecture, differentiation, and cytoprotection [7]. Hsp90-beta is also upregulated in several cancers, such as breast cancers [8]. However, the study on the expression of Hsp90-beta and its significance with lung cancer is considerably limited compared with Hsp90-alpha. In the current study, we identified the upregulation of Hsp90-beta and annexin A1 in lung cancer cells, and we further investigated the significance of this upregulation in lung cancer and the potential use of Hsp90-beta and annexin A1 as clinical markers for lung cancer.

## Methods

### Cell lines and cell culture

Human H446 small cell lung cancer (SCLC) cells and large cell lung cancer (LSCC) H520 (squamous cell carcinoma of the lung) cells were obtained from the Cell Biology Department of Medical School, Lanzhou University, China. Human A549 LAC (adenocarcinoma of the lung) cells were obtained from the Experimental Center, Medical School, Xi’an Jiaotong University. Sixteen HBE cell lines were purchased from the Tumor Cells Collection, Academy of Chinese Medical Sciences, Beijing, China. All cell lines were cultured in an RPMI 1640 medium with 10% FBS, 100 units/mL penicillin, and 100 μg/mL streptomycin (Invitrogen). The solution was maintained at 37°C in humidified 5% CO_2_ and 95% air incubator.

### Patients

Surgical tissue specimens from 96 patients with primary lung cancer who underwent surgical resection of their tumors at the Gansu Provincial Hospital, Lanzhou, Gansu, China and the second affiliated hospital, Xi'an Jiaotong University, Shaanxi, China from January 2004 to December 2010 were obtained for this retrospective study. The study followed institutional review board guidelines. Informed consent was obtained from each patient. None of the subjects received radio/chemotherapy prior to surgery. All patients who participated in this study were divided according to the tumor-node-metastasis (TNM) classification of the International Association for The Study of Lung Cancer (IASLC, Seventh edition, 2009) [9]. The tumors were histologically subtyped and graded according to the third edition of the World Health Organization guidelines. The patients were classified according to gender, and their ages ranged from 28 to 78 years (median = 56 years). Clinical characteristics were retrieved from available clinical records. The clinico-pathological factors were retrospectively assessed and are listed in Table 1. The normal control tissues consisted of two parts. Twenty-four matched adjacent non-malignant tissues were collected at sites at least 3 cm away from the edge of tumor mass. Efforts were done to avoiding contamination by the tumor cells. Twenty-two non-malignant tissues were obtained from the benign lung disease patients during lung volume reduction surgery.

**Table 1:** Clinico-pathological features of lung cancer cases (N =96)

**Group**	**Characteristics**	**Number (%)**
Sex		
	Male	73(76.04%)
	Female	23(23.96%)
Age		
	<60	54(56.25%)
	≥60	42(43.75%)
Pack years of smoking		
	>40	47(48.96%)
	20.1–40	4(4.17%)
	0.1–20	8(8.33%)
	0	37(38.54%)
Histology		
	LAC	41(42.71%)
	LSCC	39(40.63%)
	SCLC	11(11.46%)
	LCLC	3(3.13%)
	Undifferentiated	2(2.83%)
Pathologic grade		
	Poorly differentiated	26(27.08%)
	Moderately differentiated	33(34.38%)
	Well-differentiated	21(21.88%)
	Others	16(16.67%)
Clinical staging		
	IB	3(3.1%)
	IIA-IIB	53(55.3%)
	IIIA-IIIB	25(26.04%)
	IV	4(4.1%)
	Unavailable	11(11.46%)
Pleural invasion		
	Absent	82(85.42%)
	Present	14(14.58%)
Lymphatic invasion		
	Positive	55(57.29%)
	Negative	41(42.71%)

LAC, lung adenocarcinoma; LSCC, lung squamous cell carcinoma; SCLC, small cell lung cancer; LCLC, large cell lung cancer.

### Preparation and identification of cell protein samples

The cells were dissolved in a lysis buffer, and then centrifuged at 12,000 rpm for 30 min at 4°C. The supernatant was transferred to a fresh tube, and the cellular protein concentration was measured by the Bradford method. Trypsin (Promega, USA) was added to each of the groups, and equal amounts of proteins from each sample was added according to the protocol of the isobaric tags for relative and absolute quantization kit. The protein lysates of cells were labeled with the corresponding labeled reagent. The proteins were identified by 2D LC-MS /MS according to a method previously described [10]. The MS/MS spectra were collected in a data-dependent manner, in which up to four precursor ions above an intensity threshold of seven counts/s were selected for MS/MS analysis from each survey “scan.” In the tandem MS data database query, the peptide sequence tag (PKL) format files that were generated from MS/MS were imported into the Mascot search engine with an MS/MS tolerance of ± 0.05 Da to search the NCBInr database.

### Pathological studies and tissue microarray (TMA) construction

Pathologists marked morphologically representative tumor areas to construct the microarray samples and to avoid necrotic areas and areas in which cancer cells and stromal cells intermingled. The TMAs were constructed using a tissue array instrument (Beecher Instruments, Manual Tissue Arrayed, USA). A tissue core from the donor block was removed using a thin-walled needle with an inner diameter of approximately 2.0 mm. Two core samples from each tumor were precisely placed into a recipient block at specifically assigned locations. The array block was sectioned and leveled on the microscope slide, baked in an oven, and finally tested with routine H&E staining, immunohistochemistry (IHC), and *in situ* hybridization (ISH).

### IHC

The expression levels of Hsp90-beta and annexin A1 were determined using an S-P combination of IHC techniques (UltraSensitive S-P Rabbit, Product Code: SP9000, Zhongshan Jinqiao biotech company, Beijing, China). IHC was strictly implemented according to the UltraSensitive S-P Rabbit kit. The first antibody concentration consisted of a rabbit anti-human Hsp90-beta polyclonal antibody (1:100 dilution; Product Code: BA0930, Bostere Biotech Company, Wuhan, China) and the rabbit anti-human annexin A1 (1:100 dilution; Product Code: 55018-1-AP, ProteinTech Group, Inc., USA). The kit provided positive slices that served as the positive control sample, and an identical volume of PBS as a replacement to the primary antibody incubated in identical conditions was used as the negative control sample. Immunostaining was blindly evaluated by two independent experienced pathologists (Wang JS and Li J) according to a scoring method previously described [11]. At least ten randomly selected high-power fields and >1,000 cells were counted for each section. Each specimen was scored according to the intensity of staining (intensity) and the area of staining (extent). The intensity was graded according to the following scale: 0, no staining; 1+, mild staining; 2+, moderate staining; 3+, intense staining. The extent was evaluated as follows: 0, no staining of cells in any microscopic fields; 1+, <30% of tissue stained positive; 2+, between 30% and 60% stained positive; 3+, >60% stained positive. A combined staining score (intensity + extension) of ≤2, between 3 and 4, and between 5 and 6 were considered as low, moderate, and high expression levels, respectively

### ISH

The mRNA expression levels of Hsp90-beta and annexin A1 were determined by ISH. Initially, the mRNA sequences of Hsp90-beta and annexin A1 were identified in the GeneBank (MedLine, USA). The oligonucleotide probe sequences of Hsp90-beta and annexin A1 were designed using the oligonucleotide probe designing software (Vector NTI 9.0). The probe sequence of Hsp90-beta was 5′-TACCA GTGCT GCTGT AACTG AAGAA ATGCC-3′, and that of annexin A1 was 5′-TACAC CAAGT ACAGT AAGCA TGACA TGAAC AAAGT-3′. Finally, the probes were synthesized in a DNA synthesizing instrument (Bostere biotech company, Wuhan, China). ISH was strictly performed according to the ISH kit (Product Code: MK1152, Bostere biotech company, Wuhan, China). The sections were deparaffinized, rehydrated, and incubated with pepsin for 25 min at 37°C. The hybridization liquid that contains the Digoxigenin-labelled RNA probes was placed on the sections, and the sections were then covered by parafilm and incubated at 42°C for 24 h in a moisture chamber. After hybridization, the slides were washed with different concentrations of SSC to remove the excess probe. The washed slides were incubated with diluted anti-Digoxigenin antibody conjugated HRP at 37°C for 2 h at room temperature, and colored with DAB (Zhongshan Jinqiao biotech company, Beijing, China) at 37°C for 30 min with no exposure to light. The negative control samples included the following: (i) RNase treatment (20 mg/ml) hybridization and (ii) use of neither probes nor anti-Digoxigenin antibody; the controls exhibited no positive signals. The positive controls included the positive slices provided by the kit and the combined use of ISH and IHC. The mRNA expression levels of Hsp90-beta and annexin A1 were independently evaluated by two pathologists (Wang JS and Li J). The mRNA levels of Hsp90-beta and annexin A1 exhibited positive staining in the cytoplasm. A specific scoring method for ISH was performed according to a previously published report [12]. The scoring method was as follows: according to the signal intensity, the signals were divided into 4 groups, namely, absent (0), low (+), moderate (++), and high (+++). For statistical analysis, we grouped the patients as low (0, +), moderate (++), and high (+++).

### Western blot

The harvested cells were washed once with PBS, lysed with 2× sodium dodecyl sulfate (SDS)-polyacrylamide gel electrophoresis (PAGE) sample buffer (20 mM Tris, pH 8.0, 2% SDS, 2 mM dithiothreitol, 1 mM Na_3_VO_4_, 2 mM EDTA, and 20% glycerol), and boiled for 5 min. The protein concentration of each sample was determined using a Micro-BCA protein assay. In all samples, 30 μg of the total cellular protein was loaded on a 10% SDS-PAGE gel and electrophoretically separated. The proteins were transferred to polyvinylidene difluoride membranes. The membranes were blocked for 2 h at 37°C in 20 mM Tris, pH 8.0, 150 mM NaCl, and 0.05% Tween 20 (TBST) containing either 5% BSA or 5% nonfat dried milk. The membranes were incubated with various antibodies (for immunoblotting with anti-Hsp90-beta 1:200 and annexin A1 antibody 1:400) overnight at 4°C. The primary antibodies were detected using horseradish peroxidase-conjugated secondary antibodies, and after three washes with TBST, positive signals were visualized using the enhanced chemiluminescence method. All experiments were performed for three separate times.

### Statistical analysis

The associations between the expression status and clinico-pathological parameters were analyzed using the *χ*^2^, Fisher’s exact, and McNemar tests. The overall survival was measured from the date of surgery to the date of death from any cause or the date on which the patient was last known to be alive. Survival curves were plotted according to the Kaplan-Meier method and were compared using the log-rank test. A Cox proportional hazard regression model for multivariate analysis was used to test the confounding effect of the variables that are most closely associated with the expression levels of the different protein expression status. All tests were two-sided, and p-values <0.05 were considered to be statistically significant. The SPSS 15.0 software package was used to perform the statistical analysis (SPSS Institute, version 15.0, Chicago, USA).

## Results

### Identification of Hsp90-beta and annexin A1 as differential protein

Using 2D LC-MS /MS, we compared the protein expression profiles among A549, H446, and 16 HBE cells. After comparing the variations in the average abundance, a total of 26 differential proteins (C1.5-fold) in the different cells were detected and successfully identified. Two proteins were significantly upregulated in A549 cells (2.19- and 2.14-fold for Hsp90-beta and annexin A1, respectively) and also in H446 cells (1.72- and 1.67-fold for Hsp90-beta and annexin A1, respectively) compared with 16 HBE. The detailed information on Hsp90-beta and annexin A1 are listed in Table 2.

**Table 2:** Differential information of Hsp90-beta and annexin A1 between different cells identified by 2D-LC-MS/MS

**The difference between 16HBE and A549**					
**Protein ID**	**Description**	**Peptide**	**16HBE**	**A549**	**Difference (times)**
MITO:558|72222	Hsp90-beta	37	0.00	1.13	2.19
MITO:650|4502101	annexin A1	62	0.00	0.60	2.14
**The difference between 16HBE and H446**					
MITO:558|72222	**Description**	**Peptide**	**16HBE**	**NCI-H446**	**Difference (times)**
	Hsp90-beta	37	0.00	0.78	1.72
MITO:650|4502101	annexin A1	62	0.00	0.74	1.67
**The differential proteins between different cells identified by 2D-LC-MS/MS**					
MITO:558|72222	**Description**	**Protein mass**	**Protein score**	**Coverage rate**	**Difference**
	Hsp90-beta	83584.22	683.24	34.94%	p < 0.05
MITO:650|4502101	annexin A	38918.06	564.29	50.58%	

### Expressions of Hsp90-beta and annexin A1 in cancer and normal tissues

**Figure 1:**
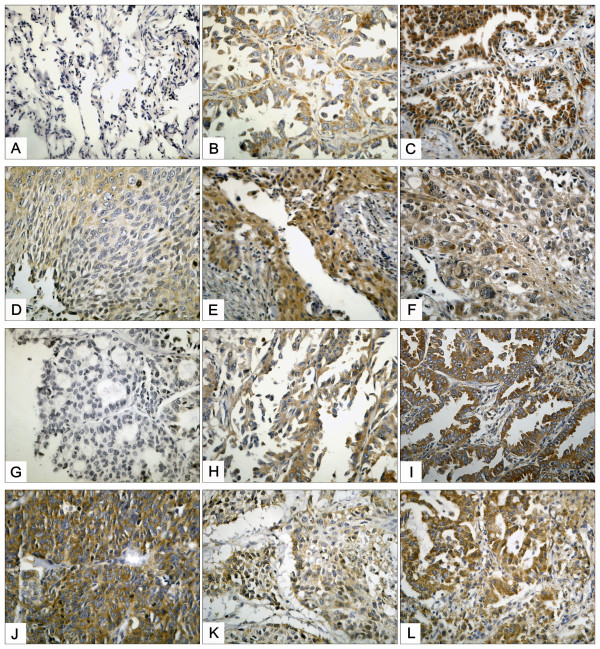
**IHC analysis of Hsp90-beta and annexin A1 in lung cancer and normal lung tissues (IHC × 400).** (**A**) Low staining of Hsp90-beta in normal tissues; (**B**) moderate staining of Hsp90-beta in moderately differentiated LAC; (**C**) high staining of Hsp90-beta in poorly differentiated LAC; (**D**) moderate staining of Hsp90-beta in moderately differentiated LSCC; (**E**) high staining of Hsp90-beta in poorly differentiated LSCC; (**F**) high staining of annexin A1 in LCLC; (**G**) low staining of annexin A1 in well-differentiated LAC; (**H**) moderate staining of annexin A1 in moderately differentiated LAC; (**I**) high staining of annexin A1 in poorly differentiated LAC; (**J**) high staining of annexin A1 in SCLC; (**K**) moderate staining of annexin A1 in moderately differentiated LSCC; (**L**) high staining of annexin A1 in poorly differentiated LSCC; LAC, adenocarcinoma of the lung; LSCC, squamous cell carcinoma of the lung; SCLC, small cell lung cancer; LCLC, large cell lung cancer.

The protein expression levels of Hsp90-beta and annexin A1 were determined by IHC in a series of 96 specimens of lung cancer tissues and a series of 46 specimens of normal tissues. Hsp90-beta and annexin A1 were highly expressed in 57 (59.4%) and 44 (45.8%) of the 96 lung cancer tissues, respectively, whereas both were lowly expressed in three (6.5%) and seven (15.2%) of the 46 normal lung tissues. The upregulation of Hsp90-beta and annexin A1 in the lung cancer tissues and the down regulation in the normal lung tissues were observed (p < 0.0005; p = 0.001) (Table 3, Figures 1A, B, C, D, E, F, G, H, I, J, K, and L). In the statistical analysis of the 24 matched cancer and normal tissues, the expression trends of Hsp90-beta and annexin A1 were consistent in all analyzed specimens (p < 0.0005; p = 0.023)

**Table 3:** Expressions of Hsp90-beta and annexin A1 in the lung cancer tissues and adjacent-cancer normal tissues

**Groups**		**N**	**Expression of Hsp90-beta**					**Expression of annexin A1**				
			**Low (%)**	**Moderate (%)**	**High (%)**	***χ***^**2**^**value**	***p*value**	**Low (%)**	**Moderate (%)**	**High (%)**	***χ***^**2**^**value**	***p*value**
All												
	Normal	46	12(26.1)	31(67.4)	3(6.5)	36.29	<0.0005	21(45.7)	18(39.1)	7(15.2)	15.05	0.001
	Cancerous	96	14(14.6)	25(26)	57(59.4)			20(20.8)	32(33.3)	44(45.8)		
Matched												
	Normal	24	7(29.17)	15(62.5)	2(8.33)	17.524	<0.0005	13(54.2)	7(29.2)	4(16.7)	7.577	0.023
	Cancerous	24	2(8.3)	6(25)	16(66.7)			4(16.7)	11(45.8)	9(37.5)		

### Correlation between the expressions of Hsp90-beta and annexin A1 and clinicopathologic factors

**Figure 2:**
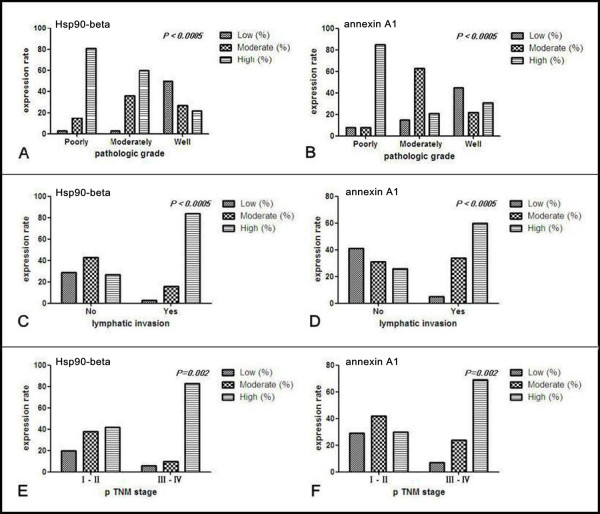
**Correlation between clinico-pathological features and the expression of Hsp90-beta and annexin A1 in lung cancer.** (**A** and **B**) Upregulation of Hsp90-beta and annexin A1 was observed in poorly differentiated lung cancer tissues compared with well-differentiated tissues (*p* < 0.0005); (**C** and **D**) Hsp90-beta and annexin A1 expressions in lung cancer cases without lymphnode metastasis was lower than that in lung cancer cases with lymph node metastasis (*p* < 0.0005); (**E** and **F**) Upregulated Hsp90-beta and annexin A1 was found in lung cancer tissues at stages III to IV compared with that at stages I to II (*p* = 0.002).

The association of several clinicopathologic factors with Hsp90-beta and annexin A1 expression is illustrated in Table 4. High expression levels of Hsp90-beta and annexin A1 were found in poorly differentiated lung cancer tissues (80.8% and 84.6%, respectively) compared with well-differentiated tissues (22.7% and 31.8%, respectively) (p < 0.0005) (Figures 2A and B). High expression levels of Hsp90-beta and annexin A1 in lung cancer cases without lymph node metastasis were both 26.8%, which is lower than what was noted in lung cancer cases with lymph node metastases as follows: N1, 85% and 60%; N2, 81.8% and 81.82%; and N3, 100% and 100%, respectively (p < 0.0005) (Figures 2C and D). Annexin A1 was significantly associated with the histological type, and was highly expressed in LAC (23/39, 59%) and SCLC (7/11, 63.6%), but lowly expressed in LSCC (12/41, 29.3%) (p < 0.05). Hsp90-beta exhibited a higher expression in SCLC (9/11, 81.82%) than in LAC (22/39, 56.4%) and LSCC (23/41, 56.1%) (p < 0.05). The expression levels of Hsp90-beta and annexin A1 in lung cancer cases of T3 to T4 were 85.7% (24/28) and 71.4% (20/28), which is higher than what was observed in lung cancer cases of T1 to T2, respectively (p = 0.001). Moreover, Hsp90-beta and annexin A1 were highly expressed in stages III (82% and 68%) and IV (100% and 75%) compared with stages I (both 0%) and II (45.3% and 32.1%) of lung cancer tissues (p < 0.05) (Figure 2E and F). However, the expressions of Hsp90-beta and annexin A1 did not correlate with other clinicopathologic factors such as gender, age, smoking imaging, and pleural invasion.

**Table 4:** Correlation between clinico-pathological features and the expressions of Hsp90-beta and annexin A1 in lung cancer

**Parameter**	**Group**	**N**	**Expression of Hsp90-beta**					**Expression of annexin A1**				
			**Low (%)**	**Moderate (%)**	**High (%)**	***χ***^**2**^**value**	***P*value**	**Low (%)**	**Moderate (%)**	**High (%)**	***χ***^**2**^**value**	***P*value**
Gender												
	Male	73	12(16.4)	22(30.1)	39(53.4)	4.49	0.105	18(24.7)	26(35.6)	29(39.7)	5.09	0.078
	Female	23	2(8.7)	3(13)	18(78.3)			2(8.7)	6(26.1)	15(65.2)		
Ages												
	<60	54	8(14.8)	13(24.1)	33(61.1)	0.251	0.882	8(14.8)	20(37)	26(48.1)	2.798	0.247
	≥60	42	6(14.3)	12(28.6)	24(57.1)			12(28.6)	12(28.6)	18(42.9)		
Smoking												
	0	37	3(8.1)	6(16.2)	28(75.7)	8.28	0.082	5(13.5)	10(27)	22(59.5)	3.856	0.248
	0.1–40	12	1(8.33)	5(41.67)	6(50)			2(16.7)	5(41.7)	5(41.7)		
	>40	47	10(21.3)	14(29.8)	23(48.9)			13(27.7)	17(36.2)	17(36.2)		
Histology												
	LAC	39	8(20.5)	9(23.1)	22(56.4)^★^	8.16	<0.05	7(17.9)	9(23.1)	23(59)^▴^	7.513	<0.05
	LSCC	41	5(12.2)	13(31.7)	23(56.1)^★^			10(24.4)	19(46.3)	12(29.3)^▴^		
	SCLC	11	1(9.1)	1(9.1)	9(81.82)^★^			2(18.2)	2(18.2)	7(63.6)^▴^		
	Others	5	0(0)	2(40)	3(60)			1(20)	2(40)	2(40)		
Pathological grade												
	Poorly	26	1(3.8)	4(15.4)	21(80.8)	31.26	<0.0005	2(7.7)	2(7.7)	22(84.6)	38.26	<0.0005
	Moderately	33	1(3.03)	12(36.36)	20(60.61)			5(15.2)	21(63.6)	7(21.2)		
	Well	22	11(50)	6(27.3)	5(22.7)			10(45.5)	5(22.7)	7(31.8)		
	Undifferentiated	15	1(6.67)	3(20)	11(73.33)			3(20)	4(26.7)	8(53.3)		
Lymphatic invasion												
	N0	41	12(29.3)	18(43.9)	11(26.8)^★★^	31.02	<0.0005	17(41.5)	13(31.7)	11(26.8)^▴▴^	19.97	<0.0005
	N1	40	1(2.5)	5(12.5)	34(85) ^★★^			2(5.5)	17(34.5)	21(60) ^▴▴^		
	N2	11	0(0)	2(18.2)	9(81.8) ^★★^			1(9.1)	1(9.1)	9(81.82)^▴▴^		
	N3	4	0(0)	0(0)	4(100) ^★★^			0(0)	0(0)	4(100) ^▴▴^		
hydrothorax												
	Absent	82	13(15.9)	23(28)	46(56.1)	2.51	0.285	18(22)	29(35.4)	35(42.7)	2.25	0.324
	Present	14	1(7.1)	2(14.3)	11(78.6)			2(14.3)	3(21.4)	9(64.3)		
T stage												
	T1 – T2	57	11(19.3)	22(38.6)	24(42.1)	14.72	0.001	17(29.8)	23(40.4)	17(29.8)	14.83	0.001
	T3 – T4	28	2(7.1)	2(7.1)	24(85.7)			1(3.6)	7(25)	20(71.4)		
	Unavailable	11	1(9.1)	1(9.1)	9(81.82)			2(18.18)	2(18.18)	7(63.64)		
pTNM												
	IB	3	1(33.3)	2(66.7)	0(0)^●^	11.449	0.022	0(0)	3(100)	0(0)^●●^	9.97	0.008
	IIA-IIB	53	10(18.9)	19(35.8)	24(45.3)^●^			16(30.2)	20(37.7)	17(32.1)^●●^		
	IIIA-IIIB	25	2(8)	3(12)	20(82)^●^			2(8)	6(24)	17(68)^●●^		
	IV	4	0(0)	0(0)	4(100)^●^			0(0)	1(25)	3(75)^●●^		
	Unavailable	11	1(9.1)	1(9.1)	9(81.82)			2(18.18)	2(18.18)	7(63.64)		
Imaging												
	Central	43	5(11.63)	15(34.88)	23(53.49)	2.68	0.261	11(20.9)	16(41.9)	16(37.2)	2.07	0.356
	Ambient	49	9(18.37)	10(24.49)	30(57.14)			8(20.4)	16(32.7)	25(46.9)		
	Unavailable	4	0(0)	0(0)	4(100)			1(25)	0(0)	3(75)		

^★^*p* < 0.05, SCLC compared with LSCC and LAC, respectively; ^▴^*p* < 0.05, LSCC compared with LAC and SCLC, respectively; ^★★^*p*<0.0005, N0 compared with N1, N2, and N3, respectively; ^▴▴^*p*<0.0005, N0 compared with N1, N2, and N3, respectively; ^●^*p* = 0.022, IB and IIA-IIB compared with IIIA-IIIB and IV, respectively; ^●●^*p* = 0.022, IB and IIA-IIB compared with IIIA-IIIB and IV, respectively; LAC, lung adenocarcinoma; LSCC, lung squamous cell carcinoma; SCLC, small cell lung cancer; LCLC, large cell lung cancer; Smoking, pack years of smoking.

### Association between mRNA and protein expressions of Hsp90-beta and annexin A1 in the matched cancer tissues and adjacent normal tissues

**Figure 3:**
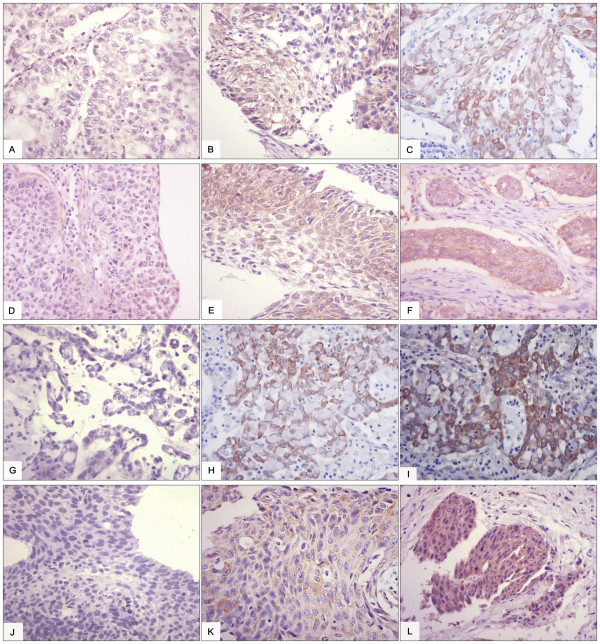
**ISH analysis of Hsp90-beta and annexin A1 mRNA in lung cancer and normal lung tissues (ISH × 400).** (**A**) Low staining of Hsp90-beta mRNA in well-differentiated LAC; (**B**) moderate staining of Hsp90-beta mRNA in moderately differentiated LAC; (**C**) high staining of Hsp90-beta mRNA in poorly differentiated LAC; (**D**) low staining of Hsp90-beta mRNA in well-differentiated LSCC; (**E**) moderate staining of Hsp90-beta mRNA in moderately differentiated LSCC; (**F**) high staining of Hsp90-beta mRNA in poorly differentiated LSCC; (**G**) low staining of annexin A1 mRNA in well-differentiated LAC; (**H**) moderate staining of annexin A1 mRNA in moderately differentiated LAC; (**I**) high staining of annexin A1 mRNA in poorly differentiated LAC; (**J**) low staining of annexin A1 mRNA in well-differentiated LSCC; (**K**) moderate staining of annexin A1 mRNA in moderately differentiated LSCC; (**L**) high staining of annexin A1 mRNA in poorly differentiated LSCC; LAC, lung adenocarcinoma; LSCC, lung squamous cell carcinoma; SCLC, small cell lung cancer; and LCLC, large cell lung cancer.

**Figure 4:**
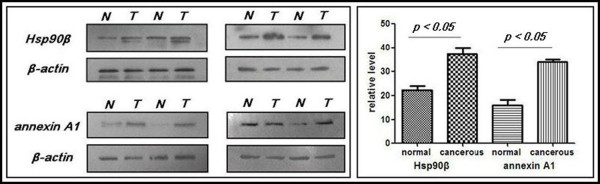
**Representative results of the Western blot of the expressions of Hsp90-beta and annexin A1 expression in the matched cancer tissues and adjacent normal tissues.** The Western blot results indicated high expression levels of Hsp90-beta and annexin A1 in the cancer tissues than the adjacent normal tissues (*p* < 0.05); N = normal tissues; T = tumor tissues.

Twenty-four matched fresh cancer tissues and adjacent normal tissues were collected from November 2010 to October 2011. The tissues were protected according to the standard process to prevent mRNA degradation. The mRNA expression levels of Hsp90-beta and annexin A1 were determined using ISH in these fresh sections. High mRNA expression levels of Hsp90-beta and annexin A1 were observed in ten (41.7%) and eight (33.3%) of the 24 lung cancer tissues, whereas both markers were lowly expressed in two (8.3%) and three (12.5%) of the 24 normal lung tissues, respectively. An upregulated mRNA expression of Hsp90-beta and annexin A1 was found in the lung cancer tissues (p = 0.006; p = 0.002) (Table 5, Figures 3 A, B, C, D, E, F, G, H, I, J, K, and L). The mRNA expressions of Hsp90-beta and annexin A1 were consistent with protein expression (McNemar test, p > 0.05). We performed Western blot to confirm the differential expressions of Hsp90-beta and annexin A1 and to verify their differential expressions in the matched cancer tissues and adjacent normal tissues. Equal protein loading was indicated by a parallel β-actin blot experiment. As shown in Figure 4, Hsp90-beta and annexin A1 were upregulated in cancerous tissues compared with normal tissues (p < 0.05) (Figure 4).

**Table 5:** The mRNA and protein expressions of Hsp90-beta and annexin A1 in matched cancer tissues and adjacent normal tissues

**Groups**		**N**	**Expression of Hsp90-beta**					**Expression of annexin A1**				
			**Low (%)**	**Moderate (%)**	**High (%)**	***χ***^**2**^**value**	***p*value**	**Low (%)**	**Moderate (%)**	**High (%)**	***χ***^**2**^**value**	***p*value**
mRNA												
	Normal	24	13(54.2)	9(37.5)	2(8.3)	10.15	0.006	15(62.5)	6(25)	3(12.5)	12.85	0.002
	Cancerous	24	4(16.7)	10(41.7)	10(41.7)			3(12.5)	13(54.2)	8(33.3)		
Protein												
	Normal	24	7(29.17)	15(62.5)	2(8.33)	17.524	<0.0005	13(54.2)	7(29.2)	4(16.7)	7.577	0.023
	Cancerous	24	2(8.3)	6(25)	16(66.7)			4(16.7)	11(45.8)	9(37.5)		

### Survival of patients with lung cancer in relation to the expressions of Hsp90-beta and annexin A1

**Figure 5:**
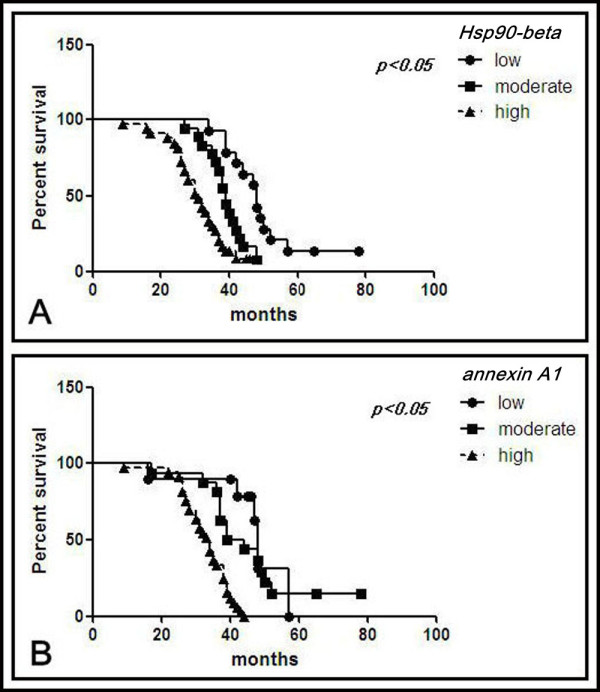
**Kaplan-Meier survival curves for positive and negative expressions of Hsp90-beta and annexin A in lung cancer.** (**A**) Among all 65 lung cancer cases, a higher expression of annexin A1 was associated with a longer post-surgery survival time (*p* = 0.014). (**B**) A higher expression of Hsp90-beta is also related to a longer post-surgery survival time (*p* = 0.021).

Overall survival was measured from the date of surgery to the date of death from any cause or the date on which the patient was last known to be alive. A total of 65 out of 96 patients had complete follow-up data based on the apparent relationship between the two markers and the clinicopathologic factors. We investigated if the expression levels could predict the clinical outcome. Statistically significant differences in disease-free survival were found, as illustrated by the Kaplan-Meier curves. Patients who exhibited high expressions of Hsp90-beta and annexin A1 had a significantly shorter post-surgical survival time prognosis compared with patients who exhibited moderate and low expressions of these markers (p < 0.05) (Figures 5A and 5B). Multivariate analysis was performed to examine the independent prognostic significance of these markers compared with the established clinical factors. The high expressions of Hsp90-beta and annexin A1 appeared to be a strong independent prognostic indicator for disease-free survival (p = 0.000 and p = 0.000, respectively), whereas pathologic grade, TNM stage, and lymphatic invasion were determined to be risk factors that decreased the post-surgical survival time (p = 0.013, p = 0.018, and p = 0.001, respectively). These values were obtained using the following risk function: H(t) = [h_0_(t)]e^(0.415X 5–1.012 X7-0.631 X8+1.552 X10+1.073X11)^ (Table 6).

**Table 6:** Cox proportional hazards regression model analysis of disease-free survival

**Variables (X)**	**Categories (different groups)**	**P value**	**OR value**		**95% CI for OR**
				**Lower**	**Upper**
Gender (X1)	Male (X_1-0_) vs. female (X_1-1_)	0.785	-	-	-
Age (*X*2)	<60 (*X*_2-0_) vs. ≥60 (*X*_2-1_)	0.492	-	-	-
Smoking (X3)	0 (X_3-0_) vs. 0.1-40 (X_3_-_1_) vs. >40 (X_3_-_2_)	1.062	-	-	-
Histology (X4)	LAC (X_4-0_) vs. LSCC (X_4_-_1_) vs. SCLC (X_4_-_2_) vs. LCLC (X_4_-_3_)	0.908	-	-	-
Differentiation (X5)	Poor (X_5-0_) vs. moderate (X_5-1_) vs. well (X_5-2_)	0.013	1.514	1.090	2.103
T stage (X6)	T1-2 (X_6-0_) vs. T3-4 (X_6-1_)	0.769	-	-	-
Lymphatic invasion (X7)	Positive (X_7-0_) vs. negative (X_7-1_)	0.018	0.697	0.516	0.941
TNM (X8)	I-II (X_8-0_) vs. III-IV (X_8-1_)	0.001	0.532	0.370	0.765
Pleural invasion (X9)	Absent (X_9-0_) vs. Present (X_9-1_)	0.154	-	-	-
Annexin A1 (X10)	Low (X_10-0_) vs. moderate (X_10-1_) vs. high (X_10-2_)	0.000	4.723	2.703	8.253
Hsp90-beta (X11)	Low (X_11-0_) vs. moderate (X_11-1_) vs. high (X_11-2_)	0.000	2.923	1.857	4.601
Imaging (X12)	Central (X_12-0_)vs. ambient (X_12-1_)	1.600	-	-	-
Risk function:		H(t) = [h_0_(t)]e^(0.415 X5 - 1.012 X7 - 0.631 X8 + 1.552 X10 + 1.073 X11)^			

LAC, lung adenocarcinoma; LSCC, lung squamous cell carcinoma; SCLC, small cell lung cancer; LCLC, large cell lung cancer; Smoking, pack years of smoking. OR, odds ratio; CI, confidence interval.

### The relative risk (RR) for the expressions of Hsp90-beta and annexin A1 in lung cancer

Pearson’s *χ*^2^-test was performed to evaluate RR associated with the expressions of Hsp90-beta and annexin A1 and lung cancer. The results indicated that the RR value for positive/negative expression of Hsp90-beta was 12.21 (p = 0.000) with a 95% confidence interval (CI) of 4.334 to 34.422. Statistical analysis results showed that subjects with higher Hsp90-beta expression exhibited a significantly higher risk for lung cancer development (RR = 12.21) compared with subjects with lower Hsp90-beta expression. The RR value of annexin A1 expression was 6.6 (p = 0.000), and the 95% CI was 2.415 to 18.04. This result indicated a higher risk for lung cancer development (RR = 6.6). The higher mRNA expression levels of Hsp90-beta and annexin A1 also indicated a higher risk for lung cancer development (RR = 16.25; RR = 13.33) compared with the protein expression (Table 7).

**Table 7:** The odds ratio for positive / negative expressions of Hsp90-beta and annexin A1 in lung cancer

**Odds ratio for**	**Risk value**	***χ***^**2**^**value**	**Fisher’s exact test (two-sided)**	**95% Confidence interval**	
				**Lower**	**Upper**
Hsp90-beta positive / negative	12.21	26.85	0.000	4.334	34.422
Hsp90-beta mRNA positive / negative	16.25	10.08	0.002	2.462	107.24
Annexin A1 positive / negative	6.6	15.09	0.000	2.415	18.04
Annexin A1 mRNA positive / negative	13.33	9.11	0.003	2.169	81.95

### The expression levels of Hsp90-beta and annexin A1 increased in the cultured human lung cancer cells

**Figure 6:**
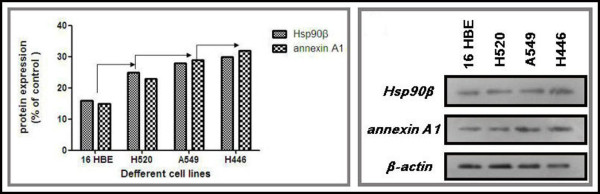
**Protein expression of Hsp90-beta and annexin A1 in cell lines using Western blot analysis.** Varied expression levels of Hsp90-beta and annexin A1 in cell levels were noted, but was generally upregulated in most lung cancer cell lines (except the H520) compared with the 16 HBE cell lines.

We examined the cultured human lung cancer cell lines for the expressions of Hsp90-beta and annexin A1. We compared these levels to those obtained from cultured cells derived from normal lung tissues. For the control cells, we used 16 HBE cell lines, which originated from the normal human bronchial epithelium. The Hsp90-beta and annexin A1 protein levels exhibited significantly upregulated expression in the A549, H520, and H446 cell lines compared with the 16 HBE cell lines. Meanwhile, a weak difference in expression was observed among the A549, H520, and H446 cell lines, which revealed that the Hsp90-beta and annexin A1 protein levels were slightly higher in the H446 and A549 cell lines compared with others, but the results was not statistically significant (Figure 6).

## Discussion

In this study, quantitative proteomic analysis was performed to identify the candidate upregulated proteins in lung cancer. Twenty-six different gene products were successfully identified as differentially expressed proteins between the lung cancer and the normal bronchial epithelial cell lines. The differential proteins are involved in various biological processes such as skeletal development, protein binding, calcium ion binding, cell motility, signal transduction, cell growth, cell-cell signaling, and glycolysis, which are all associated with cancer development and progression. Among these processes, Hsp90-beta and annexin A1 were remarkably upregulated in the lung cancer cell lines. The overexpression of Hsp90, which is the classic chaperone family in cancer, has been related to the prognosis and evolution of neoplasia similar to other Hsps. Hsp90 has two main isoforms, namely, Hsp90-alpha and Hsp90-beta. A study of various tumor cell lines revealed that Hsp90-beta was expressed in HCT116 and HeLa cells. In addition, Hsp90-beta was found in Saos-2 (osteosarcoma), SK-N-SH, HL-60 (acute promyelocytic leukemia), and A375 (malignant melanoma) cell lines [13]. Annexins are calcium and phospholipid-binding proteins that form an evolutionarily conserved multigene family, and the members of its family are widely expressed in mammals. The dysregulation of the annexin family members including annexin A1, A2, A5, A6, A7, A8, and A9, among others, were reported in numerous cancers. These markers influenced the patterns of cellular behaviors, such as cell proliferation, motility, invasiveness, and cancer-related signaling pathways, which suggest that annexins have important functions in tumor development and progression and can be used as potential biomarkers for the diagnosis and treatment of cancer [14]. Thus far, research on Hsp90-beta and annexin A1 expression patterns in lung cancer are confined to the basic research in vitro, and the expression status of lung cancer patients is rarely studied. The expressions of Hsp90-beta and annexin A1 in lung cancer clinical specimens were evaluated to determine the epidemiologic features of Hsp90-beta and annexin A1 as well as their clinicopathological significance in lung cancer. The relationships of Hsp90-beta and annexin A1 expressions with clinicopathological factors were evaluated in our study.

Our results showed that Hsp90-beta and annexin A1 exhibited a high expression in all histological types of lung cancer, particularly in poorly differentiated lung cancer. The lung cancer patients with high expressions of Hsp90-beta and annexin A1 exhibited a poorer disease-free survival than those with low expressions of Hsp90-beta and annexin A1. Thus, we can infer that high expressions of Hsp90-beta and annexin A1 can potentially promote lung cancer development. Metastasis and malignant invasion are the critical factors in the progression of lung cancer, and an alteration in the expressions of Hsp90-beta and annexin A1 is highly involved in tumor cell lymph node invasion, larger tumor size, and high TNM stage according to our study. These findings are in accordance with previous reports, where a higher level of Hsp90-beta in cancer is associated with a poor clinical outcome compared with patients with low expression levels of Hsp90-beta [15-18]. Moreover, annexin A1 was associated with metastasis and prognostic factors in multiple malignancies such as colorectal, esophageal gastric, and prostate [19-21]. This result suggests that the upregulation of Hsp90-beta and annexin A1 in the cytoplasm of tumor cells may contribute to cancer progression. The metastatic spread of tumor cells is a multi-step and complicated process. For the tumor cells to metastasize, they need to invade through the basement membrane, detach from the primary tumor mass, enter the circulation, travel to a distant secondary site, extravasate, and expand in the new environment. Each step is essential, and various proteins have critical functions in several processes. Hsp90 is essential for the stability and the function of many oncogenic client proteins, such as Her2, BCR-ABL, AKT/PKB, C-RAF, BRAF, CDK4, PLK-1, MET, mutant p53, steroid hormone receptors like androgen and oestrogen receptors, surviving, and telomerase, hTERT, VEGFR, FLT3, and hypoxia-inducible factor (HIF)-1 [22]. The inhibition of Hsp90 function causes the degradation of client proteins via the ubiquitin–proteasome pathway, which results in the depletion of multiple oncoproteins. This phenomenon causes the down regulation of signals through various oncogenic signaling pathways [23]. A number of theories on the possible signal pathway of annexin A1 in cancer development are available. Annexin A1 was shown to stimulate epithelial cell migration/invasion through the activation of formal peptide receptors in metastasis development [24]. Annexin A1 promotes metastasis formation by enhancing TGF-beta/Smad signaling and actin reorganization, which facilitates an epithelial-to-mesenchymal transition -like switch. Thus, cell migration and invasion of metastatic breast cancer cells become more efficient [25]. In the present study, Cox regression analysis results showed that high Hsp90-beta and annexin A1 expressions might be an important risk factor for the post-surgical survival time of lung cancer subjects, and that a high expression might be an unfavorable factor for the prognosis of lung cancer patients. The risk ratios for lung cancer in individuals with upregulated Hsp90-beta and annexin A1 were 12.21× and 6.6×, respectively, which are higher than those with low expressions. The final inducted variables were Hsp90-beta, annexin A1, pathologic grade, TNM stage, and lymphatic invasion. The final risk function was H(t) = [h_0_(t)]e^(0.415X 5–1.012 X7-0.631 X8+1.552 X10+1.073X11)^. Lymphatic invasion, pathologic grade, and TNM stage were also shown to be risk factors for the post-surgical survival time of lung cancer patients with OR values of 1.514, 0.697, and 0.532, respectively. The results indicated that poor differentiation and lymphatic invasion were also risk factors in reducing the survival of patients. The risk function also indicated that Hsp90-beta and annexin A1 were risk factors for lung cancer progression. These data showed that the expressions of Hsp90-beta and annexin A1 are associated with post-surgical survival time and, therefore, has the potential to become a part of the prognostic index that can predict the post-surgical survival rate of patients with lung cancer.

Annexin A1 expression was found in 59% in LAC, but 29.3% in LSCC. The degree of malignancy of LAC was significantly higher than LSCC. This result may suggest that a relationship exists between high expressions of annexin A1 and LAC. However, the mechanism remains unclear, and further investigation is required. The upregulation of Hsp90-beta and annexin A1 was observed in SCLC, but not in LSCC, LAC, and LCLC. This result suggests that the upregulation of Hsp90-beta and annexin A1 may be particularly related to the malignant invasion of SCLC. In clinical cases, early distant metastasis occurs more frequently in SCLC than in other histological types. SCLC is more aggressive and often widely metastasizes before the primary tumor mass in the lung becomes enlarged. Thus, further research is needed to explore the relationship among SCLC, Hsp90-beta, and annexin A1. Thus far, the role of annexin A1 as a prognostic factor in cancer remains ambiguous. Previous studies have reported a high level of annexin A1, but several studies reported otherwise. The function of annexin A1 apparently follows a biphasic mode during tumorigenesis, where it functions as a tumor suppressor during the early stages of the disease and as a potent stimulator of tumor progression in a late stage disease [2,26]. Hsp90-beta was more upregulated in gastric cancer tissue than in non-cancerous gastric mucosa and was also upregulated in poorly differentiated cancer tissue [27]. Hsp90-beta is overexpressed in cancer cells, and Hsp90-beta inhibitors have shown selectivity for cancer cells. Therefore, small-molecule inhibitors are being developed as anticancer therapeutics [28]. The detection of Hsp90-beta and annexin A1 showed a significant association between high expression levels and an increased risk for lung cancer. In addition, lung cancer with high levels of Hsp90-beta and annexin A1 are more likely to show an aggressive phenotype that is exemplified by a large tumor size and lymphatic metastasis. These results indicate the possibility that the levels of Hsp90-beta and annexin A1 could be risk factors for lung cancer, and can provide a new insight into the understanding of the association between Hsp90-beta, annexin A1, and lung cancer risk. The expressions of Hsp90-beta and annexin A1 in cells displayed varied levels of expressions. However, the two markers were generally upregulated in most lung cancer cell lines compared with 16 HBE cell lines, which is in accordance with previously published studies [13,29]. More importantly, the risk ratio analysis result indicates that the upregulation of Hsp90-beta and annexin A1 might be an unfavorable factor in lung cancer. The RR of Hsp90-beta and annexin A1 mRNA expression for lung cancer was higher than their proteins, with RR values of 16.25× and 13.33×, respectively. These results indicate that Hsp90-beta and annexin A1 mRNA in lung cancer exhibited the highest significance in the diagnosis and prediction of lung cancer. However, a large sample study would be required before Hsp90-beta and annexin A1 can be used as potential markers for lung cancer tumor. In addition, we performed a diagnostic test to investigate if Hsp90-beta and annexin A1 could function as indices for the pathological diagnosis in lung cancer. The sensitivity, specificity, positive predictivity, and diagnostic coincidence rate of the ability of Hsp90-beta and annexin A1 to predict lung cancer were relatively high (above of 80%), which indicates the differential diagnostic value of Hsp90-beta and annexin A1 levels for lung cancer.

However, three deficiencies in the present study exist. First, only surgical specimens were used, which results in a major patient selection bias considering that surgery is involved only in several exceptional cases possibly in stages IIIB and IV. Second, the number of study samples was relatively small, and further experimental investigations involving a larger number of samples of lung cancer patients is required to reach a more definitive conclusion. Third, we did not investigate the molecular mechanism and signal pathways of Hsp90-beta and annexin A1. Hence, RNA interference, gene transfection, and antibody neutralization should be performed to elucidate further the mutual regulation-mechanism regarding lung cancer cell lines. A detailed understanding of the function and significance of Hsp90-beta and annexin A1 is advantageous to elucidate further the biological mechanisms of lung cancer and aid in the design of preventive treatment because lung cancer is a highly malignant tumor in the respiratory system. Our preliminary results need to be confirmed by a prospective study including a large number of subjects as well as by the functional analysis of Hsp90-beta and annexin A1 through in vitro studies in the future because the number of study samples in this study is small.

## Conclusions

We demonstrated that Hsp90-beta and annexin A1 were upregulated in lung cancer, and the upregulation of these molecules in lung cancer was associated with poor post-surgical survival time and malignant tendency of lung cancer patients. These results indicate that the upregulation of Hsp90-beta and annexin A1 was potentially involved in the progression and prognosis of lung cancer. However, a larger number of lung cancer subjects is required for prospective studies, and further studies are required to investigate the potential mechanism of increased in lung cancer.

## Competing interests

The authors declare that they have no competing interests.

## Authors' contributions

Jiang XL, Cai XG, Wang JS, and Zhang M participated in the study design, discussed the results, and helped draft the manuscript. Rong BX, Yang SY, and Zhang W participated in the study design, performed experiments and data statistics, and wrote the manuscript. All authors have read and approved the final manuscript.
